# Occupational exposure risk assessment for cytotoxic drugs among clinical nurses in oncology care settings: development of a Delphi-AHP indicator system

**DOI:** 10.3389/fonc.2026.1874705

**Published:** 2026-06-30

**Authors:** Ran Hao, Mengying Yao, Yuhan Du, Huixin Jin

**Affiliations:** 1 College of Nursing and Rehabilitation, North China University of Science and Technology, Tangshan, Hebei, China; 2 Department of Pharmacy, Hebei General Hospital, Shijiazhuang, Hebei, China; 3 Hebei Key Laboratory of Clinical Pharmacy, Shijiazhuang, Hebei, China; 4 The Fifth Department of Neurology, Hebei General Hospital, Shijiazhuang, Hebei, China

**Keywords:** analytic hierarchy process, clinical nurses, cytotoxic drugs, Delphi method, indicator system, occupational exposure, risk assessment

## Abstract

**Objective:**

To develop a scientific and systematic occupational exposure risk indicator system for cytotoxic drugs (CDs) among clinical nurses and to provide an evidence-based basis for institutional risk management and targeted occupational protection strategies.

**Methods:**

This methodological study used a mixed-methods consensus-building design. Based on the work breakdown structure-risk breakdown structure (WBS-RBS) framework, an initial risk matrix was constructed through literature analysis, policy and guideline review, and semi-structured interviews. Three rounds of Delphi consultation were conducted with 16 experts in oncology nursing, nursing management, pharmacy, and occupational protection/infection control. Indicators were screened using a 5-point Likert scale, and weights were determined using the analytic hierarchy process (AHP).

**Results:**

The response rates for the three Delphi rounds were 80.00%, 93.75%, and 100.00%, respectively. The expert authority coefficients were 0.803, 0.856, and 0.891. In the third round, the coefficient of variation ranged from 0.000 to 0.264, and Kendall’s coefficient of concordance was 0.265 (P<0.01). The final indicator system included five first-level indicators, 13 second-level indicators, and 33 third-level indicators. All AHP judgment matrices met the consistency requirement (CR<0.10).

**Conclusion:**

The indicator system developed in this study integrates full-process clinical work links with multidimensional risk sources, providing a structured and quantifiable framework for early identification, prioritization, and targeted management of occupational exposure risks related to cytotoxic drugs among clinical nurses. It may serve as a practical decision-support reference for developing graded and precise occupational protection strategies, particularly in institutions without centralized intravenous admixture services.

## 1 Introduction

The global incidence of malignant tumors continues to rise. According to the latest global cancer statistics published in CA: A Cancer Journal for Clinicians, more than 20 million new cancer cases occurred worldwide in 2022 (1). Chemotherapy remains a core treatment modality, and cytotoxic drugs (CDs) are widely used because of their antitumor effects. However, their inherent genotoxic, teratogenic, and carcinogenic properties may pose serious occupational health threats to clinical nurses who handle these drugs over long periods (2).

Previous studies from Europe, North America, and other regions have shown that occupational exposure to CDs is not limited to drug compounding areas. Drug residues and potential exposure risks have been identified in infusion areas, nursing stations, patient-care areas, and other clinical environments (3, 4). Other research has indicated that administration, connection and disconnection of infusion tubing, and waste disposal may also generate occupational exposure risks (5). Multicountry investigations of guidelines and current practices for the safe handling of antineoplastic and other hazardous drugs have further shown that, although relevant guidelines have gradually improved, implementation of safe-handling measures still varies across clinical settings (6, 7). Studies in China have also monitored non-compounding clinical environments and found drug contamination in healthcare workers’ work areas and wards outside drug storage and preparation zones (8).

However, much of the existing evidence has focused on environmental monitoring, particularly objective contamination detection methods such as surface wipe sampling (4). Less attention has been paid to translating clinical workflows, exposure sources, and management weaknesses into structured and quantifiable risk assessment indicators. This gap is particularly relevant for institutions without pharmacy intravenous admixture services (PIVAS) or with incomplete centralized compounding systems, where clinical nurses may be directly involved in drug preparation, bedside administration, infusion monitoring, needle removal and catheter flushing, waste disposal, and environmental cleaning. A full-process and multidimensional occupational exposure risk assessment tool is therefore needed.

The WBS-RBS framework is a matrix-based risk identification method (9). The work breakdown structure decomposes an overall clinical workflow into specific work tasks, whereas the risk breakdown structure decomposes potential risk sources into different risk categories. By cross-mapping work tasks with risk sources, this framework can systematically identify possible occupational exposure risks at each work stage. Accordingly, this study introduced the WBS-RBS framework and combined semi-structured interviews, Delphi expert consultation, and AHP to construct an occupational exposure risk indicator system for CDs among clinical nurses, with the aim of supporting full-process risk identification, risk prioritization, and precise protection in healthcare institutions.

## 2 Materials and methods

### 2.1 Study design

This was a methodological study using a mixed-methods consensus-building design. The study consisted of five stages: literature analysis, semi-structured interviews, construction of the WBS-RBS risk matrix, Delphi expert consultation, and AHP-based weighting. First, literature retrieval, policy and guideline review, and semi-structured interviews were used to identify work links and risk sources related to clinical nurses’ contact with CDs. Second, an initial risk matrix was constructed based on the WBS-RBS framework. Third, three rounds of Delphi consultation were used to discriminate, screen, revise, and confirm the risk matrix and indicator items. Finally, AHP was used to calculate indicator weights and test matrix consistency.

### 2.2 Establishment of a multidisciplinary research team

A seven-member research team was established, including three oncology nursing experts, two researchers in hospital infection prevention and occupational protection, one pharmacy expert, and one nursing graduate student. The team was responsible for establishing the technical route and quality-control standards, reviewing the literature search strategy and interview guide, guiding construction of the WBS-RBS risk matrix, and supervising the Delphi consultation process. All team members had relevant practical experience and received pre-study training to standardize terminology and evaluation criteria.

### 2.3 Initial construction of the WBS-RBS risk matrix

#### 2.3.1 Theoretical analysis

The WBS-RBS method is a systematic risk assessment approach dominated by qualitative analysis while incorporating quantitative analytical concepts. It first subdivides the overall work process into specific work links to establish the WBS and then subdivides risks related to a given field or event to establish the RBS. A WBS-RBS matrix is then constructed to identify risks. This structured approach is suitable for developing an occupational exposure risk indicator system for CDs among clinical nurses.

#### 2.3.2 Literature search

Relevant literature on occupational exposure risks associated with CDs was searched in CNKI, Wanfang Data, CBM, PubMed, Web of Science, and other databases. National documents, policies, and guidelines were also reviewed. Potential indicator items were extracted, screened, and summarized.

#### 2.3.3 Semi-structured interviews

Based on the WBS-RBS framework, purposive sampling was used to select 15 oncology nurses from hospitals of different levels in multiple cities of Hebei Province. Their routine work involved contact with and infusion of CDs, and their professional titles ranged from junior to senior. The interview guide was developed based on literature analysis and policy/guideline review and was adapted to local clinical contexts, including PIVAS availability, whether clinical departments participated in drug preparation and bedside administration, waste disposal, and emergency handling of occupational exposure. This adaptation improved the fit between interview content and local clinical practice. The interview guide covered the following questions: (1) Does your department involve self-preparation of CDs, and what is the process? (2) What is the process for handling drug spills? (3) Do you take special protective measures during venipuncture, and have sharps injuries or drug contact occurred? (4) How are infusion devices and waste handled after needle removal, and have classification errors or unsealed disposal occurred? (5) Have you experienced psychological or physiological responses, such as poor sleep, due to long-term work in this environment?

#### 2.3.4 Construction of the WBS-RBS risk matrix

Through systematic literature review, extraction of policy and guideline information, and semi-structured interviews, the research team initially developed a work breakdown system for clinical nurses handling CDs. Their work content was summarized into five dimensions and 13 specific work indicators. Based on full-process occupational exposure analysis, risk categories were divided into five dimensions: operation process, materials and equipment, work environment, organizational management, and personnel health; 17 key risk factors were further identified. Based on this framework, an initial WBS-RBS risk matrix was constructed. In the matrix, “1” indicated that a risk factor had a direct or potential association with a corresponding work task, whereas “0” indicated that no clear or plausible association was identified. The judgment of risk presence was based on literature retrieval, semi-structured interview data, and research group discussion. Two researchers independently made the initial judgments, and disagreements were resolved through multidisciplinary team discussion. The resulting risk factor discrimination form was then submitted for the first Delphi round.

### 2.4 Delphi expert consultation

#### 2.4.1 Selection of consultation experts

Expert selection is fundamental to the scientific validity and authority of the Delphi method. The inclusion criteria were as follows: (1) professional background in clinical nursing, pharmacy, or occupational protection/infection control, with at least eight years of work experience; (2) bachelor’s degree or above and intermediate professional title or above; and (3) informed consent and willingness to participate until the end of the study. Formal invitation letters were used to explain the study purpose, consultation process, and schedule. Twenty experts were ultimately invited to participate.

#### 2.4.2 Development of the expert consultation questionnaire

The expert consultation questionnaire consisted of three parts. (i) First, the cover letter introduced the study content, consultation process, and precautions, and guaranteed confidentiality of personal information and opinions. (ii) Second, the expert consultation form was developed. The three rounds of questionnaires were not identical; rather, they were designed according to the iterative feedback and revision principles of the Delphi method. In the first round, a risk factor discrimination form based on the WBS-RBS matrix was used. Experts judged whether each potential risk factor was relevant to each work link; “1” indicated presence of a relevant risk, and “0” indicated absence of a relevant risk. This round was used to initially identify and screen potential risk factors. After the first round, the research team integrated the risk factors identified by experts and their comments to form a preliminary risk system for occupational exposure to CDs among clinical nurses. In the second round, experts rated the importance of each risk factor in this preliminary system using a 5-point Likert scale. In the third round, the questionnaire was revised based on second-round results, importance scores, and feedback; experts rated the importance of the revised items again and provided further suggestions. This round was used to finalize the occupational exposure risk system for CDs among clinical nurses. (iii) Third, the general expert information section collected basic information, familiarity with the consultation topic, and self-evaluation of judgment basis.

The Delphi questionnaire was developed based on literature analysis, semi-structured interview findings, and the WBS-RBS risk matrix. It was locally adapted by incorporating the current status of PIVAS construction, CD preparation and administration processes, waste disposal, and occupational protection management across hospitals at different levels in Hebei Province. Before formal distribution, the multidisciplinary research team reviewed and revised the questionnaire content and wording to improve clinical applicability and clarity.

#### 2.4.3 Implementation of expert consultation

Three rounds of Delphi consultation were conducted using electronic questionnaires distributed via email and WeChat. In the first round, questionnaires were sent to 20 invited experts, and 16 valid responses were received. Based on the principle of risk-priority discrimination (10), preliminary risk discrimination results were generated and are shown in **Table 1**. Based on the experts’ risk discrimination results, the research team developed the initial draft of the occupational exposure risk system for cytotoxic drugs among clinical nurses, which was then used for the second-round Delphi consultation.

**Table 1:** Risk factor discrimination matrix for occupational exposure to cytotoxic drugs among clinical nurses.

Work structure	Work task	R1a	R1b	R1c	R1d	R2a	R2b	R2c	R3a	R3b	R3c	R4a	R4b	R4c	R4d	R5a	R5b	R5c
W1	Pre-compounding preparation	1	0	0	0	1	0	0	1	0	0	0	0	0	0	0	0	0
W1	Compounding	1	0	0	0	0	0	1	1	0	0	0	0	0	0	1	0	1
W1	Post-compounding operation	0	0	1	0	0	0	0	0	1	0	0	0	0	0	0	1	0
W2	Medication verification and assessment	0	0	0	0	0	0	0	0	0	0	1	0	0	1	0	0	0
W2	Infusion device preparation	0	0	0	0	0	1	0	0	0	0	0	0	1	0	0	0	0
W2	Nurse protection preparation	0	0	0	0	1	0	0	0	0	0	0	1	1	0	0	0	1
W3	Venipuncture	0	1	0	0	1	1	0	0	0	0	0	0	0	0	1	0	0
W3	Infusion monitoring	0	0	0	1	0	1	1	0	0	0	0	0	0	0	0	0	1
W3	Changing drug solution/connectors	0	1	0	0	0	0	0	0	0	0	0	1	0	0	0	0	0
W4	Needle removal	0	1	0	1	0	0	0	0	0	0	1	0	0	0	0	0	0
W4	Patient education	0	0	0	0	0	0	0	0	0	0	0	0	0	1	0	0	0
W5	Medical waste disposal	0	0	1	0	0	0	0	0	1	0	1	1	0	0	0	0	1
W5	Environmental cleaning/disinfection	0	0	0	0	0	0	1	0	0	1	0	0	1	0	0	0	0

1 indicates the presence of a risk association; 0 indicates absence of a risk association. W1, drug preparation; W2, pre-infusion preparation; W3, intravenous administration and monitoring; W4, completion of infusion and needle removal; W5, waste disposal and environmental cleaning. R1a–R1d, drug preparation operation, administration operation, waste disposal, and emergency handling risks; R2a–R2c, personal protective equipment, infusion device, and hardware equipment risks; R3a–R3c, unclear layout zoning, contamination residue, and cleaning/disinfection risks; R4a–R4d, insufficient systems/procedures, training/assessment, resource support, and safety awareness risks; R5a–R5c, acute injury, chronic injury, and psychological stress risks.

The second-round questionnaire was distributed to the 16 experts who participated in the first round, and 15 valid responses were collected. Indicators with a mean importance score >3.50 and a coefficient of variation (CV) <0.25 were retained (11). Indicators close to the threshold were further discussed by the research team and were retained when they had clear clinical relevance or were recommended by multiple experts. The third-round questionnaire was developed accordingly.

In the third round, the questionnaire was distributed to the same 16 experts, and all 16 valid responses were received. Because the third-round results met the predefined screening criteria and expert opinions were sufficiently stable, the Delphi consultation was concluded.

### 2.5 Statistical analysis

Frequencies and percentages were used to describe expert characteristics, and questionnaire response rates were used to indicate expert engagement. Expert authority was quantified using the authority coefficient (Cr), calculated as Cr=(Ca+Cs)/2, where Ca represents the judgment coefficient and Cs represents the familiarity coefficient. Both Ca and Cs were obtained from experts’ self-evaluations in the general information questionnaire. A Cr value greater than 0.7 indicated high expert authority (12). The concentration of expert opinions was described using the mean importance score based on the 5-point Likert scale. The coordination of expert opinions was evaluated using the CV and Kendall’s coefficient of concordance (Kendall’s W), with significance testing. A smaller CV indicates higher coordination among expert opinions. Kendall’s W ranges from 0 to 1, and larger values indicate greater agreement.

After the final indicator system was established, AHP was used to calculate indicator weights and test consistency. The consistency index was calculated as CI=(λmax−n)/(n−1), where λmax is the maximum eigenvalue and n is the order of the matrix. The consistency ratio was calculated as CR=CI/RI, where RI is the average random consistency index. A CR value less than 0.10 indicated acceptable consistency. SPSS 27.0 was used to calculate mean importance scores, standard deviations, CVs, and Kendall’s W. Yaahp 10.0 was used to calculate indicator weights and AHP consistency. The significance level was set at α=0.05.

## 3 Results

### 3.1 General characteristics of experts

A total of 16 experts completed the consultation. They represented healthcare institutions at different levels in Hebei Province, including provincial, municipal, and district/county-level institutions. Regarding educational background, 12 experts (75.00%) had a bachelor’s degree, three (18.75%) had a master’s degree, and one (6.25%) had a doctoral degree. Regarding professional title, seven (43.75%) had intermediate titles, six (37.50%) had associate senior titles, and three (18.75%) had senior titles. Regarding work experience, five (31.25%) had worked for 10–20 years, six (37.50%) for 21–30 years, and five (31.25%) for more than 30 years. For work role (multiple choices allowed), seven (43.75%) were involved in nursing management, 14 (87.50%) in clinical nursing, and two (12.50%) in occupational protection/infection control.

### 3.2 Expert engagement and authority

Expert engagement was evaluated using the questionnaire response rate, valid questionnaire rate, and expert suggestion rate. Because the Delphi method is an iterative expert consultation process, the response status and expert suggestions in each round can reflect the continuity and stability of expert participation and the reliability of consultation results. In the first round, 20 experts were invited, 16 valid questionnaires were returned, and five experts provided comments. The Cr was 0.803. These 16 experts then formed the panel for subsequent rounds. In the second round, questionnaires were distributed to the same 16 experts, 15 valid responses were received, and six experts provided comments. The Cr was 0.856. In the third round, questionnaires were again distributed to the same 16 experts, all 16 valid responses were received, and three experts provided comments. The Cr was 0.891. The response rates in all three rounds exceeded 70.00%, indicating good expert engagement and authority (**Table 2**).

**Table 2:** Expert engagement and authority.

Round	Questionnaire response rate (%)	Valid questionnaire rate (%)	Expert suggestion rate (%)	Cr
Round 1	80.00	100.00	33.33	0.803
Round 2	93.75	100.00	40.00	0.856
Round 3	100.00	100.00	18.75	0.891

### 3.3 Coordination and concentration of expert opinions

In the first round, experts directly judged the presence or absence of risk factors; therefore, statistical analysis of opinion concentration and coordination was not required. In the second and third rounds, the CVs ranged from 0.084 to 0.339 and from 0.000 to 0.264, respectively. Kendall’s W values were 0.153 and 0.265, respectively (both P<0.01).

### 3.4 Establishment of the indicator system and weights

Based on the first-round expert consultation results and research group discussion, one item was added and three items were modified. The preliminary risk system was revised to include five first-level indicators, 17 second-level indicators, and 43 third-level indicators. Based on second-round expert judgments, two second-level indicators and 13 third-level indicators were deleted, and four third-level indicators were added or adjusted. For example, the second-level indicators “hardware equipment” and “cleaning and disinfection” and the third-level indicators “lack of personal protection during venipuncture” and “failure to use biological safety cabinets and closed transfer devices” were deleted. The item “inconvenient access to emergency supplies or equipment failure” was divided into “inconvenient access to and operation of exposure emergency supplies” and “missing or faulty emergency supplies and equipment.” The item “high detection rate of surface contamination by cytotoxic drugs in the work environment” was revised to “lack of surface contamination monitoring in the work environment or failure to rectify excessive results,” and “low self-reporting rate of acute symptoms” was revised to “failure to self-report acute symptoms.” The indicator system was then revised to include five first-level indicators, 13 second-level indicators, and 33 third-level indicators.

In the third round, expert opinions became stable, no further comments were made on the item framework, and all items were adopted into the final risk system. Based on third-round feedback, the third-level indicator “improper emergency handling of drug leakage” was revised to “non-standardized emergency handling of drug leakage.” The final system included five first-level indicators, 13 second-level indicators, and 33 third-level indicators. All judgment matrices met the consistency requirement (CR<0.10) (13). Indicator weights calculated according to expert opinions are shown in **Table 3**.

**Table 3:** Consultation results for the occupational exposure risk indicator system for cytotoxic drugs among clinical nurses.

Risk indicator	Importance	CV	Weight
1 Operation process risk	4.79 ± 0.13	0.03	0.342
1.1 Drug preparation operation risk	4.75 ± 0.00	0.00	0.061
1.1.1 Failure to prepare and inspect personal protective equipment (PPE)	4.75 ± 0.45	0.09	0.031
1.1.2 Failure to use standardized anti-splash techniques	4.75 ± 0.45	0.09	0.031
1.2 Administration operation risk	4.75 ± 0.45	0.09	0.031
1.2.1 Non-standardized procedures related to changing drug solutions	4.56 ± 0.51	0.11	0.029
1.2.2 Failure to wear gloves during needle removal and catheter flushing/sealing	4.69 ± 0.48	0.10	0.032
1.3 Waste disposal risk	4.92 ± 0.14	0.03	0.093
1.3.1 Non-standardized sharps disposal	5.00 ± 0.00	0.00	0.032
1.3.2 Non-standardized classification of contaminated waste	5.00 ± 0.00	0.00	0.032
1.3.3 Unqualified waste packaging and handling	4.75 ± 0.45	0.09	0.031
1.4 Emergency handling risk	4.86 ± 0.09	0.02	0.126
1.4.1 Non-standardized emergency handling of drug leakage	4.94 ± 0.25	0.05	0.032
1.4.2 Non-standardized reporting, assessment, and follow-up after sharps injuries	4.94 ± 0.25	0.05	0.032
1.4.3 Inconvenient access to and operation of emergency supplies	4.81 ± 0.40	0.08	0.031
1.4.4 Missing or expired emergency supplies and equipment	4.75 ± 0.45	0.09	0.031
2 Materials and equipment risk	4.88 ± 0.09	0.02	0.126
2.1 Risk related to the use of personal protective equipment	4.81 ± 0.18	0.04	0.062
2.1.1 Failure to wear double gloves in a standardized manner	4.94 ± 0.25	0.05	0.032
2.1.2 Non-standardized wearing of protective clothing	4.69 ± 0.48	0.10	0.030
2.2 Infusion device risk	4.94 ± 0.09	0.02	0.064
2.2.1 Failure to use closed infusion devices	5.00 ± 0.00	0.00	0.032
2.2.2 Failure to assess leakage caused by infusion devices	4.88 ± 0.34	0.07	0.032
3 Work environment risk	4.75 ± 0.00	0.00	0.092
3.1 Work area risk management	4.75 ± 0.00	0.00	0.092
3.1.1 Unreasonable functional zoning management of the preparation room	4.75 ± 0.45	0.09	0.030
3.1.2 Lack of surface contamination monitoring in the work environment or failure to rectify excessive results	4.75 ± 0.45	0.09	0.031
3.1.3 Non-standardized access management for personnel entering the preparation room	4.75 ± 0.45	0.09	0.031
4 Organizational management risk	4.56 ± 0.29	0.06	0.326
4.1 Low completeness of systems and procedures	4.80 ± 0.06	0.01	0.124
4.1.1 Absence of standard operating procedures (SOPs) for key links	4.75 ± 0.45	0.09	0.031
4.1.2 Incomplete emergency plans and response procedures	4.81 ± 0.40	0.08	0.031
4.1.3 Delayed updating of management systems	4.75 ± 0.45	0.09	0.031
4.1.4 Excessive nursing workload	4.86 ± 0.34	0.07	0.032
4.2 Insufficient training and assessment risk	4.78 ± 0.04	0.01	0.061
4.2.1 Lack of special safety training	4.75 ± 0.45	0.10	0.031
4.2.2 Lack of training-effect evaluation and feedback mechanisms	4.81 ± 0.40	0.08	0.031
4.3 Risk related to insufficient resource support	4.46 ± 0.57	0.13	0.086
4.3.1 Insufficient safety protection facilities and supplies	4.69 ± 0.48	0.10	0.032
4.3.2 Insufficient full-time or qualified personnel	4.88 ± 0.34	0.07	0.032
4.3.3 Insufficient special budget support for occupational safety	3.81 ± 0.91	0.24	0.025
4.4 Weak safety awareness risk	4.19 ± 0.80	0.19	0.054
4.4.1 Insufficient awareness among nurses regarding safety training	4.75 ± 0.45	0.09	0.031
4.4.2 Failure of nurses to meet assessment standards for knowledge of occupational exposure hazards associated with cytotoxic drugs	3.63 ± 0.96	0.26	0.023
5 Personnel health risk	4.39 ± 0.51	0.12	0.113
5.1 Physical health risk	4.75 ± 0.18	0.04	0.061
5.1.1 Failure to self-report acute symptoms in a timely manner	4.88 ± 0.34	0.07	0.032
5.1.2 Failure to intervene when individual skin barrier damage occurs	4.63 ± 0.50	0.11	0.030
5.2 Mental health risk	4.03 ± 0.13	0.03	0.052
5.2.1 Failure to regularly assess work stress, such as anxiety and depression, among nurses	3.94 ± 0.85	0.22	0.025
5.2.2 Failure to provide psychological counseling and related support for staff	4.13 ± 0.72	0.17	0.027

## 4 Discussion

### 4.1 Scientific validity and reliability of the risk indicator system

This study used a mixed qualitative and quantitative approach to construct an occupational exposure risk indicator system. The WBS-RBS framework is widely used in risk management (14), but its application in medical occupational exposure risk assessment remains relatively limited. By structurally decomposing clinical work processes and risk sources, this framework helps avoid the limitations of identifying risks from a single procedure or a single contamination outcome. On this basis, the research team analyzed policies and guidelines from different regions and countries, reviewed relevant domestic and international literature, and conducted semi-structured interviews with nurses from clinical departments at different hospital levels to identify work content and exposure risk links more comprehensively. Semi-structured interviews strengthened the clinical relevance of the indicators, Delphi consultation ensured professionalism and consensus in indicator screening, and AHP quantified indicator weights. Therefore, this study combined qualitative identification with quantitative weighting, strengthening the scientific validity and reliability of the indicator system in terms of theoretical basis, methodological process, and presentation of results.

The expert consultation questionnaire was developed based on literature analysis, semi-structured interview findings, and the WBS-RBS risk matrix. It was adapted to the local context of hospitals at different levels in Hebei Province, including PIVAS availability, CD preparation and administration processes, waste disposal, and occupational protection management. Before formal consultation, the multidisciplinary research team reviewed and revised the questionnaire content and wording to improve clinical applicability. The response rates in all three rounds exceeded 70%, and all Cr values were greater than 0.7, indicating good expert engagement and authority. In the third round, Kendall’s W was 0.265, indicating moderate rather than strong agreement; the CV ranged from 0.000 to 0.264, and Kendall’s W was statistically significant. These findings suggest that although complete agreement was not achieved, an acceptable consensus was formed. The moderate level of agreement may be related to the broad scope of the indicator system and the multidisciplinary background of the expert panel. Experts in clinical nursing, nursing management, pharmacy, infection control, and occupational protection may emphasize different risk dimensions. For example, clinical nursing experts may focus more on direct operational risks such as administration, needle removal, sharps injuries, and waste disposal, whereas management and protection experts may emphasize systems and procedures, resource support, training assessment, and environmental monitoring. Thus, moderate agreement is reasonable in a multidimensional occupational exposure risk assessment.

### 4.2 Content analysis of the risk indicator system

From the perspective of indicator weights and content, operation process risk and organizational management risk occupied important positions in this system. This suggests that preventing occupational exposure to CDs among clinical nurses depends not only on standardized clinical operations but also on institutional systems, training, resource allocation, and management support. Previous studies have shown that the administration stage carries relatively high exposure risk, especially during infusion-line connection and disconnection and waste management (5). This is consistent with the second-level indicators “administration operation risk,” “waste disposal risk,” and “emergency handling risk” in this study. The corresponding third-level indicators further specify concrete risk points, including changing drug solutions, needle removal and catheter flushing/sealing, sharps disposal, contaminated waste classification, and emergency handling of drug leakage.

Organizational management risk also had a high weight in this study, indicating that CD exposure prevention cannot rely only on individual nurse compliance. Healthcare institutions also need to support prevention through system development, material supply, training and assessment, and continuous quality improvement. Previous research has identified insufficient training, weak safety awareness, and inconsistencies between guidelines and clinical practice as major barriers to safe chemotherapy administration (15). Other studies have reported that insufficient PPE supply and insufficient awareness of occupational exposure hazards among healthcare workers may increase exposure risk (16, 17). These findings correspond to the organizational management indicators in this study, including systems and procedures, training and assessment, resource support, and safety awareness.

Some indicators had relatively low mean importance scores or relatively high CV values, such as “insufficient special budget support for occupational safety,” “failure of nurses to meet assessment standards for knowledge of occupational exposure hazards associated with cytotoxic drugs,” and “failure to regularly assess work stress, such as anxiety and depression, among nurses.” These results may reflect differences in experts’ perceptions of direct and indirect exposure risks. Compared with drug leakage, sharps injuries, and other risks that may directly lead to exposure events, budget support, knowledge assessment, and psychological stress evaluation influence occupational exposure more indirectly and over a longer period. Therefore, some experts may have assigned relatively lower scores or held more divergent opinions. Nevertheless, these indicators have clear clinical management significance. Special budget support affects the allocation of PPE, closed infusion devices, emergency supplies, and training resources; insufficient knowledge assessment may weaken nurses’ understanding of CD hazards and protective requirements; and lack of psychological stress assessment may undermine occupational health maintenance in long-term exposure settings. Therefore, after expert group discussion, these indicators were retained to strengthen exposure risk prevention from organizational management and personnel health perspectives.

### 4.3 Feasibility and practical application of the risk indicator system

The risk indicator system developed in this study can serve as a standardized and structured operational tool for occupational exposure risk assessment in healthcare institutions. In practice, institutions may establish a multidisciplinary assessment team composed of nursing managers, clinical nurses, pharmacists, infection control personnel, and occupational protection staff. The team can evaluate each indicator through on-site observation, document review, staff interviews, occupational exposure records, training and assessment records, emergency drill records, and environmental monitoring results. Based on indicator weights, institutions can calculate comprehensive risk scores for different work links, identify high-risk processes and priority intervention items, and formulate graded protective measures. Assessments can be conducted periodically or after changes in workflows, staffing, or equipment conditions to support dynamic risk monitoring and continuous quality improvement.

The WBS-RBS model used in this study is not intended to replace traditional environmental monitoring methods such as surface wipe sampling; rather, it provides methodological complementarity. Wipe sampling can provide objective evidence of drug contamination in the work environment, but its results are often constrained by sampling timing, drug type, sampling site, and testing conditions. It therefore mainly reflects contamination status under specific temporal and spatial conditions and cannot fully reveal system-level process risks. In contrast, the WBS-RBS framework structurally decomposes work processes and risk sources, enabling proactive identification of potential risk points and providing a systematic basis for intervention strategies. In clinical application, surface wipe sampling results can be incorporated as important evidence for the work-environment risk dimension within this indicator system. At the same time, the WBS-RBS framework can support systematic, comprehensive, and dynamic control of occupational exposure risks associated with CDs, thereby improving occupational protection management.

### 4.4 Limitations and future research directions

This study has several limitations. First, the consultation experts were all from healthcare institutions in Hebei Province. The geographic scope was limited, and the indicator system was developed primarily through expert consensus. Multicenter clinical studies have not yet been conducted to externally validate the reliability, validity, predictive performance, and practical operability of the system; the questionnaire and assessment tool have also not undergone full standardization testing. These factors may limit the generalizability of the system. Second, the study focused only on clinical nurses and did not include oncologists, pharmacists, pharmacy intravenous admixture service personnel, or other healthcare workers who also handle CDs. The coverage of occupational groups was therefore limited. Third, the level of expert agreement in the Delphi consultation was moderate, indicating that experts from different professional backgrounds still differed in their understanding and judgment of some risk indicators.

Future research should conduct multicenter, large-sample studies across different regions and hospital levels to verify and optimize this indicator system. The study population should also be expanded to include multiple categories of healthcare workers exposed to CDs, so that a more comprehensive occupational exposure risk assessment system can be developed.

## 5 Conclusion

This study integrated the WBS-RBS framework, literature analysis, semi-structured interviews, Delphi expert consultation, and AHP weighting to identify and evaluate occupational exposure risks associated with CDs within a full-process and multidimensional framework. The resulting indicator system helps address fragmented risk identification and insufficient management dimensions in previous studies and provides a structured risk identification tool for institutions without PIVAS or with incomplete PIVAS systems. Because this study was limited by the research period, large-sample empirical research has not yet been conducted to validate the reliability and validity of the system. Future studies should apply this risk system in departments using CDs across hospitals of different levels and evaluate its predictive performance, clinical usefulness, sensitivity, and feasibility through prospective cohort studies or cross-sectional investigations.

## Data Availability

The original contributions presented in the study are included in the article/supplementary material. Further inquiries can be directed to the corresponding author.

## References

[1] Bray F Laversanne M Sung H Ferlay J Siegel RL Soerjomataram I . Global cancer statistics 2022: GLOBOCAN estimates of incidence and mortality worldwide for 36 cancers in 185 countries. CA Cancer J Clin. (2024) 74:229–63. doi: 10.3322/caac.21834 38572751

[2] Ramazani M Jaktaji RP Shirazi FH Tavakoli-Ardakani M Salimi A Pourahmad J . Analysis of apoptosis related genes in nurses exposed to anti-neoplastic drugs. BMC Pharmacol Toxicol. (2019) 20:74. doi: 10.1186/s40360-019-0372-0 31791417 PMC6889625

[3] Poupeau C Tanguay C Plante C Gagné S Caron N Bussières JF . Pilot study of biological monitoring of four antineoplastic drugs among Canadian healthcare workers. J Oncol Pharm Pract. (2017) 23:323–32. doi: 10.1177/1078155216643860 27084515

[4] Walton AL Powell MA Ledbetter L Bush MA . A scoping review of surface wipe sampling for antineoplastic drug contamination in patient care areas. J Occup Environ Hyg. (2025) 22:495–514. doi: 10.1080/15459624.2025.2471397 40305371

[5] Bernabeu-Martínez MÁ Sánchez-Tormo J García-Salom P Sanz-Valero J Wanden-Berghe C . Perception of risk of exposure in the management of hazardous drugs in home hospitalization and hospital units. PloS One. (2021) 16:e0253909. doi: 10.1371/journal.pone.0253909 34197532 PMC8248625

[6] The State Council of the People’s Republic of China . Notice on issuing the Health China Action-Implementation Plan for Cancer Prevention and Control (2023-2030) (2023). Available online at: https://www.gov.cn/zhengce/zhengceku/202311/content_6915380.htm (Accessed June 19, 2026).

[7] Mathias PI MacKenzie BA Toennis CA Connor TH . Survey of guidelines and current practices for safe handling of antineoplastic and other hazardous drugs used in 24 countries. J Oncol Pharm Pract. (2019) 25:148–62. doi: 10.1177/1078155217726160 28841099 PMC7895469

[8] Mei XH Zhang YF Lin YR Huang HJ Li J Huang XH . Environmental exposure assessment of occupational exposure to antineoplastic drugs among healthcare workers. Chin J Prev Med. (2022) 23:845–50.

[9] Wang HD Liu H Bai XY Chen XY Wang ZW Wang LP . Full life-cycle risk assessment and control of gas cylinders in university laboratories. Res Explor Lab. (2025) 44:249–57.

[10] Li LW . Risk management research on the undercutting of the existing line by Beijing Metro Line 16. Beijing: Beijing Jiaotong University (2019).

[11] Cai LB Liu YJ Guo YR Wang Q Zhan YX Liu YY . Construction of orthopedic rehabilitation nursing quality evaluation indicators. Chin J Nurs. (2021) 56:508–14. doi: 10.3761/j.issn.0254-1769.2021.04.005

[12] Cai WW Zhang XX Ye MS Luo YX Ruan WQ Li J . Construction of nursing-sensitive quality indicators for patients undergoing colonoscopy based on the structure-process-outcome model. J Nurs. (2023) 30:1–6.

[13] Luo R Zhang C Liu Y . Health risk assessment indicators for the left-behind elderly in rural China: a Delphi study. Int J Environ Res Public Health. (2020) 17:340. doi: 10.3390/ijerph17010340 31947818 PMC6981890

[14] Weiyang J Xiaoliang HE Du H . Safety risk analysis of large-scale complex project based on WBS-RBS and interval numbers. Int J Plant Eng Manage. (2022) 27:129–43.

[15] Quispe Condor YS García Saavedra LE Rodríguez Zambrano JE Espinoza Acuña MB Bedoya Ticlavilca OG . Standards for the safe administration of chemotherapy in oncological patients 2015-2020: a systematic review [Estándares para la administración segura de quimioterapia en pacientes oncológicos 2015-2020: Revisión Sistemática. J Global Health Med. (2021) 5:50–65. doi: 10.32829/ghmj.v5i2.155

[16] Kim O Lee H Jung H Jang HJ Pang Y Cheong HK . Korean nurses’ adherence to safety guidelines for chemotherapy administration. Eur J Oncol Nurs. (2019) 40:98–103. doi: 10.1016/j.ejon.2019.04.002 31229212

[17] Hao ML Bian Y Zhu JQ Chen M Chen L Yan JF . Investigation and analysis of occupational exposure to antineoplastic drugs among healthcare workers in Sichuan Province. China Pharm. (2020) 31:1009–14.

